# Outcomes of prehospital traumatic cardiac arrest managed by helicopter emergency medical service personnel in Japan: a registry data analysis

**DOI:** 10.1186/s12245-023-00550-9

**Published:** 2023-10-12

**Authors:** Hiroki Nagasawa, Kazuhiko Omori, Ken-ichi Muramatsu, Ikuto Takeuchi, Hiromichi Ohsaka, Kouhei Ishikawa, Youichi Yanagawa

**Affiliations:** Department of Acute Critical Care Medicine, Shizuoka Hospital, Juntendo University, 1129 Nagaoka, Izunokuni City, Shizuoka 410-2295 Japan

**Keywords:** Helicopter emergency medical service, Mortality, Prehospital, Registry data, Traumatic cardiac arrest

## Abstract

**Background:**

Traumatic cardiac arrest (TCA) is associated with poor outcomes. Helicopter emergency medical services (HEMSs) are often used to transport critically ill patients to hospitals. However, the role of HEMS in the treatment of TCA remains unclear. Therefore, in this study, we aimed to determine the current status of patients with prehospital TCA managed by HEMS personnel in Japan and compare the outcomes of patients who experienced TCA before and after the arrival of HEMS.

**Methods:**

The Japanese Society for Aeromedical Services registry data of patients managed by HEMS personnel from April 2015 to March 2020 were analyzed in this retrospective cohort study. HEMS arrival and physicians’ interventions at the scene were the variables of interest. The survival rate and neurological outcomes at 28 days after injury were analyzed.

**Results:**

Of the 55 299 registered patients, 722 who experienced prehospital TCA were included in the analysis. The distribution of first-witnessed TCA was as follows: pre-emergency medical service (EMS) arrival (*n* = 426/722, 60.3%), after EMS arrival (*n* = 113/722, 16.0%), and after HEMS arrival (*n* = 168/722, 23.8%). The 28-day survival rate was 6.2% (*n* = 44/706), with a cerebral performance category of 1 or 2 in 18 patients. However, patients who experienced TCA after receiving interventions provided by physicians before HEMS arrival had the worst outcomes, with only 0.6% of them surviving with favorable neurological outcomes. Multivariable analysis revealed that securing the intravenous route by the EMS team (adjusted odds ratio: 2.43, 95% confidence interval [CI]: 1.11–5.30) and tranexamic acid infusion by the HEMS team (adjusted odds ratio: 2.78, 95% CI: 1.16–6.64) may have increased the return of spontaneous circulation (ROSC) rate.

**Conclusions:**

The results of our study were similar to those reported in previous studies with regards to the use of HEMS in Japan for transporting patients with TCA. Our findings suggest that in patients with severe trauma, cardiac arrest after initiation of HEMS, the highest level of prehospital medical intervention, may be associated with an inferior prognosis. Tracheal intubation and administration of tranexamic acid by the EMS team may increase the rate of ROSC in TCA.

## Background

Traumatic cardiac arrest (TCA) is a severe condition that occurs in injured patients. Despite medical advancements worldwide, TCA still has a high mortality rate, especially in prehospital settings [1]. In a recent Japanese study published in 2021, the survival rate of patients with prehospital TCA was 4.5% [2]. Helicopter emergency medical services (HEMSs) are often used to transport critically ill patients to hospitals. However, the role of HEMS personnel in the treatment of TCA remains unclear.

In Japan, the HEMS team includes physicians. Several global studies have shown the efficacy of HEMS in improving the outcomes of severely injured patients with trauma, [3, 4] and similar results have been reported in Japan [5, 6]. Some studies have also investigated the use of HEMS [7–9] or physician interventions [10] for managing prehospital TCA. However, to our knowledge, no study has reported on the status of patients with prehospital TCA managed by the HEMS team in Japan based on registry data.

On the basis of our clinical experience, we observed that patients who experienced prehospital TCA after HEMS interventions did not survive at our institutions. This led us to hypothesize that the timing of the arrest could be crucial for the survival of patients with TCA. Patients who experience cardiac arrest after the physicians initiate medical interventions might be less likely to survive; however, no evidence supports this hypothesis.

This study aimed to assess the current status of patients with prehospital TCA managed by the HEMS personnel in Japan and compare our results with those reported in previous studies. Moreover, we aimed to test our hypothesis that patients with trauma who experienced TCA after HEMS arrival would have worse outcomes than those who experienced TCA before the HEMS arrival.

## Methods

### Study design and setting

To evaluate the current status of patients with prehospital TCA managed by the HEMS personnel in Japan and compare our results with those reported in the literature, this retrospective nationwide cohort study used the registry data from the Japanese Society for Aeromedical Services (JSAS-R). The JSAS-R registered the data of patients treated by the HEMS personnel between April 2015 and March 2020. This study was approved by the Medical Ethics Committee of Juntendo University Shizuoka Hospital (approval number: R-733), conducted in accordance with the Declaration of Helsinki, and adhered to the Strengthening the Reporting of Observational Studies in Epidemiology guidelines [11].

As of April 2020, 53 helicopters cover each area in Japan during the daytime only (for 365 days); the HEMS team does not operate at night. At all institutions, the HEMS team consists of 1–2 physicians and 1–2 nurses. All HEMS are dispatched by emergency medical services (EMSs), and the HEMS crew almost always works with the EMS crew. The EMS teams consist of 3–4 staff and can request HEMS at any time, before or after arrival at the scene. The EMS staffs can only provide limited medical interventions to injured patients, such as securing intravenous access, epinephrine injection (only performed while in cardiac arrest), and endotracheal intubation (only performed while in cardiac arrest) [12]. On the basis of the HEMS dispatch standards established by the Japanese Society for Aeromedical Services, there is no clear description of “traumatic cardiac arrest;” it is only described as “severe trauma.” In Japan, none of the patients with TCA who are managed by the HEMS team are declared dead in the field.

### Selection of participants

We examined the data of patients with TCA who were judged to have experienced cardiac arrest at least once before hospital arrival. Patients with injuries caused by drowning, burns, or toxins were excluded from the study. Patients with an unclear survival status on admission were also excluded.

### Measurements

We collected the following information of patients with trauma who experienced prehospital cardiac arrest registered in the JSAS-R: age, sex, date, time from injury to arrival at the hospital, medical interventions (EMS crew: securing intravenous access, epinephrine injection, and endotracheal intubation; HEMS crew: drug injection [vasopressors, sedations, muscle relaxants, hemostatic agents, opioids, and painkillers], endotracheal intubation, resuscitative thoracotomy, and blood transfusion), timing of the cardiac arrest, abbreviated injury scale (AIS) score, return of spontaneous circulation (ROSC) on admission, 28-day mortality, and cerebral performance category (CPC) on day 28. In this study, ROSC means the patient was admitted to the hospital alive. The collected data were anonymized by the supervisors.

### Outcomes and analysis

Using the data extracted from the JSAS-R, the prehospital TCA cohort was divided into two groups: patients with ROSC and patients without ROSC. For between-group comparison, the baseline characteristics and nonconvertible factors were analyzed. Logistic regression analysis of the following variables of medical interventions and patients’ status was performed to analyze the odds of ROSC incidence: age ≥ 75 years, sex, EMS intervention (securing the intravenous route, epinephrine dose, and tracheal intubation), and HEMS intervention (vasopressor agent dose, hemostatic agent dose, tracheal intubation, chest drainage, resuscitative thoracotomy, blood transfusion, and on-scene time ≥ 20 min).

Next, to assess the patients’ outcome according to the time of first-witnessed TCA, the cohort was divided into three groups: before EMS arrival (pre-EMS phase), after EMS arrival (EMS phase), and after HEMS arrival until hospital arrival (HEMS phase).

Categorical data are expressed as numbers and percentages (%). Continuous variables are expressed as means and standardized differences (SDs) for normally distributed data, and as the medians and interquartile ranges (IQRs) for non-normally distributed data. The Mann–Whitney U test or Kruskal–Wallis test was used to analyze continuous data, while the Fisher exact test or chi-square test was used to analyze categorical data. All statistical analyses were performed using EZR 1.54 (Saitama Medical Center, Jichi Medical University, Saitama, Japan), a graphical user interface for R 4.0.2 (The R Foundation for Statistical Computing, Vienna, Austria) [13]. Statistical significance was set at a *p*-value of < 0.05 or based on the 95% confidence interval (CI).

## Results

### Characteristics of the study samples

In total, 55 299 patients were registered in the JSAS-R, and 27 811 patients were documented as having sustained trauma, of whom 814 experienced TCA. After exclusion (drowning: 6, burns: 25, toxins: 2, unknown survival status: 59), only 722 patients were included in the final analysis. The characteristics of the study sample are presented in Table 1. The median (IQR) age was 66 (46–78) years, and 113 of the 487 patients (23.2%) were women. Most patients sustained blunt injuries (*n* = 440/489, 90.0%), with minor penetration of the trauma agent (*n* = 33/489, 6.7%). The distributions of first-witnessed cases of TCA were as follows: pre-EMS (*n* = 426/722, 60.3%), EMS arrival (*n* = 113/722, 16.0%), and HEMS arrival (*n* = 168/722, 23.8%). Approximately 21.2% (*n* = 153/722) of the patients achieved ROSC, and the 28-day survival rate was 6.2% (*n* = 44/706); of the 44 patients who survived, 18 achieved CPC 1 or 2.

**Table 1:** Baseline characteristics of patients who experienced traumatic arrest and were managed by the HEMS crew

Characteristics^a^	*n* = 722	Missing, n (%)
Age, median (IQR), year	66 (46–78)	233 (32.2)
Sex, n (%)		235 (32.5)
Male	374 (76.8)	
Female	113 (23.2)	
Type of injury, n (%)		233 (32.2)
Blunt	440 (90.0)	
Traffic accident	258 (58.6)	
Fall	141 (32.0)	
Other	41 (9.3)	
Penetrating	33 (6.7)	
Other	16 (3.3)	
First witness of cardiac arrest, n (%)		15 (2.1)
Pre-EMS phase	426 (60.3)	
EMS phase	113 (16.0)	
HEMS phase	168 (23.8)	
Outcome, n (%)		
ROSC	153 (21.2)	0 (0.0)
28-day survival rate	44 (6.2)	16 (2.2)
28-day CPC 1 or 2	18 (2.6)	21 (2.9)
28-day CPC 1 or 2 rate	18/42 (42.8)	2/44 (4.5)

*CPC* cerebral performance category, *EMS* emergency medical service, *HEMS* helicopter emergency medical service, *IQR* interquartile range, *ROSC* return of spontaneous circulation, *SD* standardized difference

^a^All categorical variables are presented as counts and percentages (%), and all numerical variables are expressed as the means and standardized differences (SDs) or medians and interquartile ranges (IQRs)

### Main findings

Table 2 shows the characteristics and outcomes of the ROSC (–) (*n* = 569) and ROSC ( +) (*n* = 153) groups. The ROSC (–) group had higher AIS scores for the limbs (median [IQR]: 0 [0, 3] vs. 2 [0, 3], *p* = 0.04) than the ROSC ( +) group. With regard to the medical interventions provided by the HEMS teams, the ROSC ( +) group received significantly less chest drainage insertion (23.0% versus [vs.] 7.8%, *p* < 0.001), vasopressor agent use (77.5% vs. 62.7%, *p* < 0.001), endotracheal intubation (78.8% vs. 69.3%, *p* = 0.02), and resuscitative thoracotomy than the ROSC (–) group (34.3% vs. 13.1%, *p* < 0.001). Additionally, hemostatic agent use (7.6% vs. 13.7%, *p* = 0.02) was significantly higher in the ROSC ( +) group than in the ROSC (–) group. No significant differences were observed in the interventions provided by the EMS teams.

**Table 2:** Characteristics of the ROSC (–) versus ROSC ( +) patients with traumatic prehospital cardiac arrest

Characteristics^a^	ROSC (–)^b^ (*n* = 569)	ROSC ( +)^b^ (*n* = 153)	*p*-value^c^
Age, median (IQR), years	67 (47, 79)	61.5 (43, 75)	0.10
Sex, n (%)			0.69
Male	296 (76.3)	78 (78.8)	
Female	92 (23.7)	21 (21.2)	
Type of injury, n (%)			0.24
Blunt	350 (90.4)	90 (88.2)	
Penetrating	27 (7.0)	6 (5.9)	
Others	10 (2.6)	6 (5.9)	
Time, median (IQR), minute			
HEMS call ~ HEMS contact patients	21 (17, 28)	23 (18, 30)	0.43
HEMS stay at scene	22 (16, 27)	22.5 (16, 28)	0.64
HEMS call ~ hospital arrival	53 (45, 66)	54 (46, 66)	0.62
AIS, median (IQR)			
Head (*n* = 281)	2 (0, 5)	1 (0, 4)	0.53
Face (*n* = 279)	0 (0, 0)	0 (0, 0)	0.50
Chest (*n* = 284)	4 (0, 5)	3 (0, 5)	0.94
Abdomen (*n* = 290)	0 (0, 2)	0 (0, 3)	0.52
Limbs (*n* = 288)	0 (0, 3)	2 (0, 3)	0.04
Skin (*n* = 282)	0 (0, 1)	0 (0, 1)	0.27
Interventions, n (%)			
EMS crew			
Intravenous route	97/538 (18.0)	31/144 (21.5)	0.34
Epinephrine	57/536 (10.6)	11/142 (7.7)	0.35
Endotracheal intubation	17/535 (3.2)	6/145 (4.1)	0.60
HEMS crew			
Chest drainage	131/569 (23.0)	12/153 (7.8)	< 0.001
Vasopressor agents^d^	441/569 (77.5)	96/153 (62.7)	< 0.001
Bicarbonate	4/569 (0.7)	4/153 (2.6)	0.07
Hemostatic agents^e^	43/569 (7.6)	21/153 (13.7)	0.02
Endotracheal intubation	445/565 (78.8)	106/153 (69.3)	0.02
Resuscitative Thoracotomy	195/569 (34.3)	20/153 (13.1)	< 0.001
Blood transfusion	10/569 (1.8)	2/153 (1.3)	1.00

*AIS* abbreviated injury scale, *EMS* emergency medical service, *HEMS* helicopter emergency medical service, *IQR* interquartile range, *ROSC* return of spontaneous circulation

^a^Categorical variables are presented as counts and percentages (%), whereas numerical variables are presented as medians and interquartile ranges (IQR)

^b^Fifty-nine out of 781 cases had missing data for ROSC

^c^The results were statistically significant based on *p* < 0.05

^d^Vasopressor agents included epinephrine, norepinephrine, phenylephrine, and dopamine

^e^Hemostatic agents included tranexamic acid and carbazochrome sodium sulfonate

**Fig. 1:**
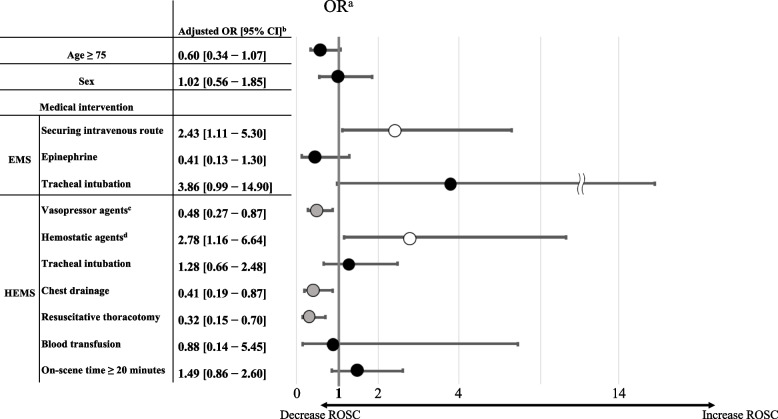
Odds ratios for the ROSC. ^a^Odds ratios of each prehospital factor for the ROSC. The horizontal bars indicate the 95% CIs. The thick vertical line represents an OR of 1.0, indicating no significant difference. The white circles on the horizontal bars mean a significantly higher OR for the ROSC, the gray circles mean a significantly lower OR, and the black circles mean an OR with no significant difference. ^b^The results were significant based on the 95% CI. ^c^Vasopressor agents included epinephrine, norepinephrine, phenylephrine, and dopamine. ^d^Hemostatic agents included tranexamic acid and carbazochrome sodium sulfonate. EMS: emergency medical service, HEMS: helicopter emergency medical service, OR: odds ratio, ROSC: return of spontaneous circulation, CI: confidence interval

In multivariable analysis, securing the intravenous route by the EMS team (adjusted odds ratio [OR]: 2.43, 95% CI: 1.11–5.30) and tranexamic acid infusion by the HEMS team (adjusted OR: 2.78, 95% CI: 1.16–6.64) were the factors that increased the ROSC rate, whereas the administration of vasopressor agents by the HEMS team (adjusted OR: 0.48, 95% CI: 0.27–0.87), chest drainage (adjusted OR: 0.41, 95% CI: 0.19–0.87), and resuscitative thoracotomy (adjusted OR: 0.32, 95% CI: 0.15–0.70) decreased the ROSC rate (Fig. 1). Moreover, the other interventions had no significant effects.

Table 3 shows the characteristics and outcomes of the patients in the EMS and HEMS phases. The HEMS phase group were older (median [IQR]: pre-EMS phase vs. EMS phase vs. HEMS phase = 61 [40, 74] years vs. 69 [53, 81] years vs. 72.5 [60, 82] years, *p* < 0.001) and had a higher proportion of female patients than the other phase groups (17.9% vs. 27.8% vs. 31.4%, *p* = 0.007). The HEMS phase group also had higher AIS scores for the chest (median [IQR]: 4 [0, 5] vs. 3 [0, 3.5] vs. 4 [1.5, 5], *p* = 0.02) and abdomen than the other phase groups (0 [0, 1] vs. 0 [0, 0] vs. 2 [0, 3], *p* < 0.001). The rates of medical interventions, including securing the intravenous route (pre-EMS vs. EMS vs. HEMS; 22.7% vs. 18.3% vs. 9.7%, *p* = 0.002) and epinephrine injection (13.5% vs. 9.3% vs. 2.4%, *p* < 0.001), performed by the EMS crew were significantly higher in the EMS phase groups than in the HEMS phase group. Concerning the use of interventions, the rates of sedation (2.8% vs. 3.5% vs. 29.8%, *p* < 0.001) and muscle relaxant therapy (1.4% vs. 1.8% vs. 18.5%, *p* < 0.001) were significantly higher in the HEMS phase group than in the EMS phase groups. Significant differences were observed in the rates of the following variables among the three phases: chest drainage insertion (17.8% vs. 15.9% vs. 28.0%, *p* = 0.01), vasopressor agent use (85.2% vs. 84.1% vs. 41.1%, *p* < 0.001), hemostatic agent use (5.9% vs. 16.8% vs. 11.3%, *p* = 0.001), endotracheal intubation by the HEMS crews (78.7% vs. 79.6% vs. 68.3%, *p* = 0.02), and resuscitative thoracotomy (35.9% vs. 31.9% vs. 13.7%, *p* < 0.001). The rates of ROSC (26.1% vs. 29.2% vs. 4.8%, *p* < 0.001), 28-day survival (7.2% vs. 11.0% vs. 1.2%, *p* = 0.002), and CPC 1 or 2 at 28 days (2.4% vs. 6.5% vs. 0.6%, *p* = 0.01) were higher in the EMS phase groups than in the HEMS phase group.

**Table 3:** Characteristics of patients with prehospital traumatic cardiac arrest in each phase

Characteristics^a^	Pre-EMS phase (*n* = 426)	EMS phase (*n* = 113)	HEMS phase (*n* = 168)	*p*-value^b^
Age, median (IQR), year	61 (40, 74)	69 (53, 81)	72.5 (60, 82)	< 0.001
Sex, n (%)				0.007
Male	229 (82.1)	57 (72.2)	81 (68.6)	
Female	50 (17.9)	22 (27.8)	37 (31.4)	
Type of injury, n (%)				0.25
Blunt	241 (88.9)	74 (87.1)	111 (94.1)	
Penetrating	18 (6.6)	9 (9.6)	5 (4.2)	
Others	12 (4.4)	2 (2.1)	2 (1.7)	
Time, median (IQR), minute				
HEMS call ~ HEMS contacts patients	22 (18, 28)	22 (17, 28)	22 (17, 28)	0.90
HEMS stay at scene	21 (16, 26)	22.5 (17, 29)	23 (18, 29)	0.03
HEMS call ~ hospital arrival	52 (44, 64)	55 (47, 66)	53 (45, 70)	0.26
AIS, median (IQR)				
Head (*n* = 281)	2 (0, 4)	1 (0, 4)	3 (0, 5)	0.21
Face (*n* = 279)	0 (0, 0)	0 (0, 0)	0 (0, 0)	0.88
Chest (*n* = 284)	4 (0, 5)	3 (0, 3.5)	4 (1.5, 5)	0.02
Abdomen (*n* = 290)	0 (0, 1)	0 (0, 0)	2 (0, 3)	< 0.001
Limbs (*n* = 288)	0 (0, 3)	1 (0, 3)	1 (0, 3)	0.11
Skin (*n* = 282)	0 (0, 1)	0 (0, 1)	0 (0, 1)	0.09
Interventions, n (%)				
EMS crew				
Intravenous route	91/401 (22.7)	20/109 (18.3)	16/165 (9.7)	0.002
Epinephrine	54/401 (13.5)	10/107 (9.3)	4/164 (2.4)	< 0.001
Endotracheal intubation	18/399 (4.5)	4/109 (3.7)	1/165 (0.6)	0.07
HEMS crew				
Chest drainage	76/426 (17.8)	18/113 (15.9)	47/168 (28.0)	0.01
Vasopressor agents^c^	363/426 (85.2)	95/115 (84.1)	69/168 (41.1)	< 0.001
Bicarbonate	6/426 (1.4)	1/113 (0.9)	1/168 (0.6)	0.68
Hemostatic agents^d^	25/426 (5.9)	19/113 (15.9)	19/168 (11.3)	0.001
Sedation	12/426 (2.8)	4/113 (3.5)	50/168 (29.8)	< 0.001
Muscle relaxant	6/426 (1.4)	2/113 (1.8)	31/168 (18.5)	< 0.001
Opioids	3/426 (0.6)	0/113 (0.0)	4/168 (2.4)	0.09
Endotracheal intubation	333/423 (78.7)	90/113 (79.6)	114/167 (68.3)	0.02
Resuscitative thoracotomy	153/426 (35.9)	36/113 (31.9)	23/168 (13.7)	< 0.001
Blood transfusion	7/426 (1.6)	1/113 (0.9)	4/168 (2.4)	0.63
Outcome, n (%)				
ROSC	111/426 (26.1)	33/113 (29.2)	8/168 (4.8)	< 0.001
28-day survival rate	30/414 (7.2)	12/109 (11.0)	2/168 (1.2)	0.002
28-day CPC 1 or 2^e^	10/410 (2.4)	7/107 (6.5)	1/167 (0.6)	0.01
28-day CPC 1 or 2 rate	10/30 (33.3)	7/12 (58.3)	1/2 (50.0)	0.35
28-day CPC				0.01
CPC 1	7/411 (1.7)	7/107 (6.5)	0/167 (0.0)	
CPC 2	3/411 (0.7)	0/107 (0.0)	1/167 (0.6)	
CPC 3	6/411 (1.5)	2/107 (1.9)	0/167 (0.0)	
CPC 4	9/411 (2.2)	2/107 (1.9)	1/167 (0.6)	
CPC 5	386/411 (93.7)	96/107 (89.8)	165/167 (98.8)	

*AIS* abbreviated injury scale, *CPC* cerebral performance category, *EMS* emergency medical service, *HEMS* helicopter emergency medical service, *IQR* interquartile range, *ROSC* return of spontaneous circulation

^a^Categorical variables are presented as counts and percentages (%), whereas numerical variables are presented as medians and interquartile ranges (IQRs)

^b^The results were statistically significant based on *p* < 0.05

^c^Vasopressor agents included epinephrine, norepinephrine, phenylephrine, and dopamine

^d^Hemostatic agents included tranexamic acid and carbazochrome sodium sulfonate

## Discussion

This study highlights two important points that distinguish it from other studies. First, to our knowledge, this report is the first to demonstrate that patients with trauma who experience TCA after physician interventions in the prehospital scenario are likely to have poor outcomes. Second, unlike previous studies, [1, 3, 4, 7–10, 14–18] this study used the registry data contributed by all HEMS institutions in Japan, thereby minimizing the deviations or biases in the patients’ characteristics and backgrounds.

### Mortality

In a recent review [1] of prehospital TCA cases, the mortality rate observed was 96.2% (95% CI: 95.0–97.2); when only the registry data were considered, the mortality rate was 97.2% (95% CI: 96.3–98.0). The rate of favorable neurological outcomes (CPC 1 or 2, or Glasgow Outcome Scale score 4 or 5) was 35.8%. Although it is challenging to provide direct comparisons with these results, our study showed a favorable mortality rate (93.8%) and favorable neurological outcomes (40.9%). Thus, the benefit of HEMS for TCA at the prehospital stage seemed consistent with the results of previous studies.

### Phases of prehospital TCA

Prehospital TCA occurs in three phases. The first phase (the pre-EMS phase) occurs immediately after the patient is injured before the arrival of the EMS crew. In our study, the majority (60.3%) of prehospital TCA events occurred during this phase. However, the occurrence of TCA in the pre-EMS and EMS phases is not directly linked to poor outcomes. On the basis of our results, patients who experienced TCA in the pre-EMS and EMS phases had better prognoses than those who had TCA in the HEMS phase. During the prehospital TCA phase, patients who experienced TCA in the EMS phase had the most favorable outcome when the TCA was witnessed after the EMS team arrived and before the arrival of the HEMS team. This finding is consistent with that reported by Kitano et al. [19]. The most severe cases of prehospital TCA occurred in the HEMS phase, consistent with our clinical experience. However, one speculation is that the limited space and fewer personnel in the helicopter may hinder the provision of adequate chest compression and treatment for the patients during transport. From a different point of view, it might also be argued that the means to save the patient when cardiac arrest occurs in a situation where the highest prehospital intervention, the intervention of the physician, is limited.

### EMS interventions

In Japan, the EMS crew has a limited ability to provide care to patients with trauma before cardiac arrest; previous studies have shown that securing intravenous access did not improve the outcomes of patients with traumatic shock [12, 20]. However, Katayama et al. [20] reported that fluid administration by EMS reduced the incidence of cardiopulmonary arrest upon hospital arrival. The present study supports the hypothesis that intravenous access established by the EMS crew can increase the rate of ROSC. This might be related to the better rate of ROSC in the pre-EMS and EMS phases. Although epinephrine is a drug that the EMS crew can administer to patients with cardiac arrest, previous studies [14, 15, 19] and our study suggest that it may not improve the outcomes of patients with trauma.

### Tranexamic acid

Tranexamic acid, a drug that has not been previously studied in this context, may be effective against prehospital TCA in the future [21]. In a prehospital trauma care setting, not in patients with TCA, a recent study reported that tranexamic acid did not improve long-term neurological outcomes; however, it reduced 24-h mortality [22]. Although this study was not performed in patients with TCA, our study similarly showed the potential and efficacy of tranexamic acid in TCA. The EMS crews are not yet permitted to administer tranexamic acid in Japan; hence, further research is required.

### Endotracheal intubation

Previous studies have reported conflicting evidence regarding the survival benefits of prehospital tracheal intubation for prehospital TCA [9, 16]. A recent review [1] found that tracheal intubation did not significantly affect the outcomes of patients with prehospital TCA, which was consistent with the findings of our study. However, the rate of sedative and muscle relaxant administration was higher during the HEMS phase, which may have contributed to the development of cardiac arrest. Although these drugs may be necessary for controlling agitation after achieving ROSC, they should be avoided during prehospital intubation of patients with severe trauma to prevent cardiac arrest. Moreover, bag-valve-mask ventilation is effective in prehospital situations; therefore, physicians should not necessarily prioritize the performance of tracheal intubation over this alternative [9].

### Resuscitative thoracotomy

Resuscitative thoracotomy is the most invasive procedure; although some studies have suggested that it may be effective for penetrating patients with trauma who are experiencing cardiac arrest [17, 23], others have reported poor outcomes associated with this procedure [18]. Our study supports the latter finding. In Japan, blunt trauma is more common than penetrating trauma, and there is limited evidence to support the use of resuscitative thoracotomy in patients with blunt trauma [24, 25]. Therefore, this procedure should not be performed in patients who experienced TCA without careful consideration of the underlying mechanism of injury.

### Blood transfusion

Blood transfusion can improve the outcomes of patients with TCA [9]. However, it was not observed in the present study. This may be because prehospital blood transfusion is not yet widely performed by HEMS in Japan.

### Limitations

This study had some limitations. First, there was a considerable amount of missing data in the JSAS-R, especially for TCA situations, which made the analysis difficult. Second, data on the detailed timing of the cardiac arrest and ROSC, first monitored rhythm, and presence of bystander cardiopulmonary resuscitation were not registered in the JSAS-R database. Third, there was a potential for patient selection bias when using the registry data. Fourth, as this was a retrospective study, the causality between patient outcomes and medical interventions could not be proven. Fifth, this database did not register the timing of interventions, so we could not identify whether the medical interventions had been performed as resuscitation, performed after resuscitation, or performed for other purposes. Finally, the causes of TCA were not identified.

## Conclusions

Regarding the use of HEMS in Japan for transporting patients with TCA, the results of our study were consistent with those reported in previous studies. Our findings suggest that patients who experienced TCA after HEMS arrival had worse outcomes than those who arrested before EMS arrival or after EMS arrival. Our study highlights the importance of carefully considering the timing and circumstances of cardiac arrest in severe trauma patients during HEMS interventions. Additionally, our study revealed that the procedures of tracheal intubation by EMS teams and the administration of tranexamic acid may increase the rate of ROSC for patients with TCA. Understanding these associations can help improve prehospital care and potentially lead to better patient outcomes.

## Data Availability

The datasets used and/or analyzed during the current study are available from the corresponding author on reasonable request.

## Abbreviations

AIS: Abbreviated injury scale
CPC: Cerebral performance category
EMS: Emergency medical services
JSAS-R: Japanese Society for Aeromedical Services
HEMS: Helicopter emergency medical services
IQR: Interquartile range
ROSC: Return of spontaneous circulation
SD: Standardized difference
TCA: Traumatic cardiac arrest
